# Low Probability of Intercept-Based Radar Waveform Design for Spectral Coexistence of Distributed Multiple-Radar and Wireless Communication Systems in Clutter

**DOI:** 10.3390/e20030197

**Published:** 2018-03-16

**Authors:** Chenguang Shi, Fei Wang, Sana Salous, Jianjiang Zhou

**Affiliations:** 1 Key Laboratory of Radar Imaging and Microwave Photonics, Ministry of Education, Nanjing University of Aeronautics and Astronautics, Nanjing 210016, China; 2 School of Engineering and Computing Sciences, Durham University, Durham DH1 3DE, UK

**Keywords:** radar waveform design, signal-to-clutter-plus-noise ratio (SCNR), mutual information (MI), low probability of intercept (LPI), spectral coexistence, distributed multiple-radar system (DMRS)

## Abstract

In this paper, the problem of low probability of intercept (LPI)-based radar waveform design for distributed multiple-radar system (DMRS) is studied, which consists of multiple radars coexisting with a wireless communication system in the same frequency band. The primary objective of the multiple-radar system is to minimize the total transmitted energy by optimizing the transmission waveform of each radar with the communication signals acting as interference to the radar system, while meeting a desired target detection/characterization performance. Firstly, signal-to-clutter-plus-noise ratio (SCNR) and mutual information (MI) are used as the practical metrics to evaluate target detection and characterization performance, respectively. Then, the SCNR- and MI-based optimal radar waveform optimization methods are formulated. The resulting waveform optimization problems are solved through the well-known bisection search technique. Simulation results demonstrate utilizing various examples and scenarios that the proposed radar waveform design schemes can evidently improve the LPI performance of DMRS without interfering with friendly communications.

## 1. Introduction

### 1.1. Background and Motivation

In recent years, distributed multiple-radar system (DMRS) has already been shown to have a number of potential advantages over monostatic radar owing to its spatial and signal diversities [1,2]. Due to the unique structure of the multiple-radar system, several diverse and independent waveforms can be simultaneously emitted by different transmitters [3]. Thus, DMRS can be employed to detect and track targets for defence purposes and is on a path from theory to practical use.

Traditionally, radar and wireless communication systems utilize the frequency band in an exclusive fashion, which are widely separated in the radio frequency (RF) spectrum such that they do not generate any harmful interference to the other [4,5,6]. However, with the rapid development of wireless communications and services with high bandwidth requirements, there is RF spectrum scarcity in commercial mobile communications such that a large number of radio spectrum designated for radar systems are underutilized [6].

Currently, spectrum sharing has received considerable attention [7], which enables radar and wireless communication users work in the same frequency band to make full use of RF spectrum resource. For instance, Global System for Mobile Communications (GSM) systems overlap with high ultra high frequency (UHF) radar systems, and WiMax and Long Term Evolution (LTE) systems partially overlap with S-band radars. The main problem is the harmful interference that one system exerts to the other. On the one hand, the interference generated by the communication system reduces the probability of detection. On the other hand, the interference power can reduce the capacity of a communication system. As such, various spectrum sharing approaches such as dynamic spectrum sensing and management, waveform optimization and power control are adopted to minimize interference effects, whose aim is to enable radar and communication systems to share the RF spectrum efficiently. In [8], the performance of wideband communication systems coexisting with narrowband interference is investigated, where the closed-form expressions for bit-error probability of a spread-spectrum systems are calculated. Aubry et al. in [9] study the synthesis of waveforms optimizing radar performance while satisfying several spectral compatibility constraints, where a polynomial computational complexity algorithm based on semidefinite relaxation and randomization is proposed. In [10], the RF spectrum congestion problem is also investigated, which presents the optimization theory-based radar waveform design method for spectrally dense environments. The authors in [11] develop a mathematical framework for spectrum sharing in networks consisted of narrowband and ultra-wide band wireless systems. Furthermore, a tutorial on stochastic geometry-based modeling and analysis is proposed for cellular networks [12], which can be applied for other wireless networks that impose interference protection around sensor nodes. In [13], a novel mechanism is proposed for spectral coexistence between radar and orthogonal frequency division multiplexing (OFDM) communication systems, which optimally assigns the subcarriers based on the importance of each channel. The work in [14] develops a dynamic spectrum allocation method for the coexistence between a radar system and a communication system, which jointly optimizes the transmitted waveform and RF spectrum for a given signal-to-interference-plus-noise ratio (SINR). Bica et al. study the problem of time delay estimation for coexisting multicarrier radar and communication systems [15]. It is shown that radar can improve the target estimation performance by exploiting the communication signals scattered off the target in a passive way. Reference [6] formulates the optimization problem of joint transmit designs for coexistence of multiple-input multiple-output (MIMO) wireless communications and sparse sensing radars in clutter, where the SINR of radar system can be enhanced by optimizing the MIMO radar transmit precoder and the communication transmit covariance matrix with a specified rate requirement for the communication system. In [16], some novel bounds on performance of the joint radar and communication system are defined. More recently, the authors in [17] proposed a new framework for pulsed radars and communication systems in coexistence.

Although the aforementioned studies provide us a guidance to deal with the problem of radar and communication systems in coexistence, they are all addressed solely for the monostatic radar. For the DMRS case, the limitations and calculations are more complicated. To the best of our knowledge, the problem of low probability of intercept (LPI)-based radar waveform design in signal-dependent clutter has not been considered in DMRS and communication coexistence literature until now.

### 1.2. Relation to the Literature

Since LPI design is an essential and challenging part of military operations in modern electronic warfare, it is of high importance to adaptively control the radar transmit resources to reduce its emitted energy while guaranteeing a desired target detection/characterization performance [18]. Technically speaking, low transmission power, large revisit interval, short dwell time, and ultra-low sidelobe antenna will enhance the LPI performance of radar system [19,20,21,22]. In [22], two LPI based radar waveform optimization criteria are presented to minimize the total transmitted energy of DMRS for a given SINR and MI threshold, respectively, whereas the effects of communication interference signals on the LPI based radar waveform design results are ignored. In [23], the optimal radar waveform design algorithm is provided with the communication signals acting as interference to the monostatic radar system. The authors in [24,25,26] maximize the probability of detection and mutual information (MI) by optimally designing OFDM radar waveform with a minimum capacity constraint for communication system, respectively. It is also confirmed that the radar performance can be significantly improved by employing the scattering off the target due to communication signals at the radar receiver. However, neither the DMRS scenario nor the power minimization based radar waveform design is considered in [23,24,25,26]. Additionally, the work in [27] investigates the problem of LPI based OFDM radar waveform design in signal-dependent clutter and white Gaussian noise for joint radar and communication system, whereas it is assumed that the exact perfect knowledge of target spectra (target spectrum is the Fourier transform of target impulse response) is known, which is impossible to capture in practice. Furthermore, in [28], power minimization based robust OFDM radar waveform optimization is explored for spectral coexisting radar and communication systems in clutter and colored noise, where the target spectra lie in uncertainty sets bounded by known upper and lower bounds. Simulation results show that utilizing the communication signals scattered off the target can remarkably reduce the total transmitted power of radar system. However, the previous system model and derivations are not suitable for the DMRS case.

### 1.3. Major Contributions

In view of the aforementioned problems, in this paper, we present novel LPI-based radar waveform design strategies for spectral coexistence of distributed multiple-radar and wireless communication systems in clutter by building on the previous results in [22,23,27,28]. The DMRS consisting of multiple radars coexists with a communication system in the same frequency band. The proposed approaches optimize the transmission waveform of each radar with the communication signals acting as interference to the radar system, in order to minimize the total transmitted energy while enabling DMRS to meet certain target detection/characterization performance. The task of DMRS is target tracking in clutter, that is, the target parameters obtained from previous tracking cycles are available for the following tracking cycle to optimize the transmission waveforms for better LPI performance. In practice, the clutter parameters can be estimated when the target is absent, while the power spectral density (PSD) of communication signal is also known at the radar receiver after a previous estimation step [28]. Therefore, some target and environment parameters, such as the target position, the target spectra with respect to different radars, the PSD of signal-dependent clutter, and the PSD of communication signal are assumed known a priori.

The major contributions of the proposed work are summarized as follows:

(1) The problem of LPI-based radar waveform design for the coexisting distributed multiple-radar and wireless communication systems in clutter is investigated. Mathematically speaking, the LPI-based radar waveform design is a problem of minimizing the total transmitted energy of DMRS by optimizing the transmission radar waveform of each radar for a predetermined target detection/characterization requirement, while minimizing the effects to the friendly communication system. It is first assumed that the radar receivers know the exact perfect knowledge of the target spectra, the PSDs of clutter and communication signal, and the propagation losses of corresponding paths. To gauge the system performance, the signal-to-clutter-plus-noise ratio (SCNR) [22,23,27,29] and MI between the received echo and the target impulse response [22,28,29,30,31] are then derived to characterize the target detection and estimation performance, respectively. Subsequently, the SCNR- and MI-based optimal radar waveform design strategies are proposed.
(2) Though the computation capability of fusion center grows exponentially thanks to techniques such as cloud computing and integrated circuits, the optimal radar waveform design involves high computational complexity. In this paper, the SCNR- and MI-based optimal radar waveform design strategies are solved analytically, and the bisection search method is exploited to find the optimal solutions for the formulated problems. It is shown that significant computational savings can be obtained through the utilization of bisection algorithm when compared to the exhaustive search approach [22].
(3) Numerical results are provided to demonstrate that the LPI performance of DMRS can evidently be improved by employing the proposed radar waveform design schemes. It is also shown that the transmit energy allocation is determined by the target spectra and the PSD of communication waveform. That is to say, we should concentrate more transmit energy for the radar that has a large target spectrum and suffers less communication interference.

### 1.4. Outline of the Paper

The rest of this paper is organized as follows: Section 2 introduces the considered system model when the distributed multiple-radar and communication systems coexisted. We then provide the underlying assumptions needed in this paper. In Section 3.1, the basis of the LPI-based radar waveform design for multiple-radar and communication systems in coexistence is presented. The SCNR- and MI-based radar waveform design strategies are proposed in Section 3.2 and Section 3.3 respectively, where the resulting problems of radar waveform design are solved by the bisection research technique. The performance of the presented strategies is assessed in detail via modeling and simulation in Section 4, whose superiority in terms of LPI performance compared to uniform waveform design method is illustrated via comparative numerical results. Finally, we present our concluding remarks in Section 5.

*Notation:* The continuous time-domain signal is denoted by s(t); The Fourier transform of s(t) is S(f). The symbol ∗ signifies the convolution operator. The superscript ${\left(\cdot \right)}^{T}$ and ${\left(\cdot \right)}^{*}$ indicate transpose and optimality.

## 2. System and Signal Models

### 2.1. Problem Scenario

We consider a coexistence scenario, where a DMRS consisting of $N_{t}$ radars and a wireless communication base station (BS) operate utilizing the same carrier frequency. Without loss of generality, we restrict our analysis to the single communication BS scenario. However, the model and the derivations can easily be extended to $N_{c}$ communication BSs [23,27].

**Figure 1:**
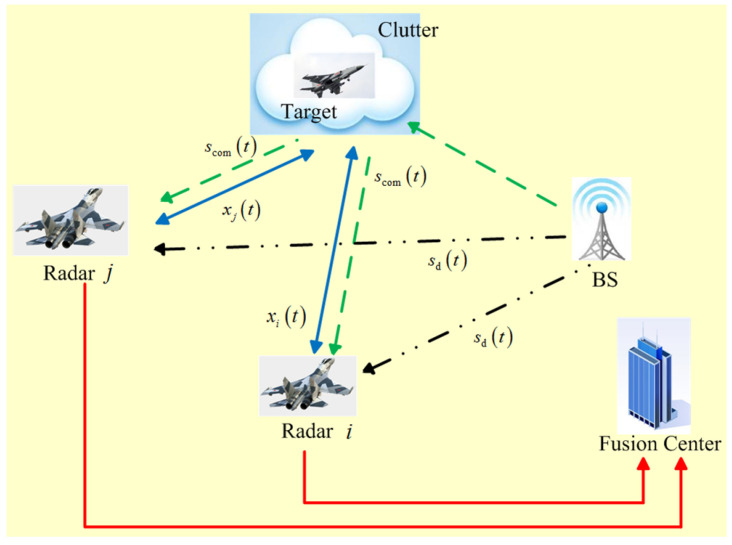
Illustration of the system model for spectral coexistence between multiple-radar and wireless communication systems.

Such a system for spectral coexistence between a DMRS and a wireless communication BS with a target is illustrated in Figure 1. The primary objective of DMRS coexisting with a communication system is to minimize the total transmitted energy by optimizing the transmission waveform of each radar, which is constrained by a desired target detection/characterization performance requirement. It is worth mentioning that the designed radar waveforms should not interfere with the friendly communication system and also minimize the interference effect of the communication signals on radar’s target detection/characterization performance. The *i*-th radar receives the echoes scattered off the target and the signal-dependent clutter due to its transmitted signal $x_{i}\left(t\right)$ as well as the signal from the communication BS whose location is supposed to be known. The communication signal $s_{\mathrm{com}}\left(t\right)$ is received via three paths: two paths which are due to the scattering off the target and the signal-dependent clutter, and a line of sight path $s_{d}\left(t\right)$. It is assumed that the PSD of communication signal $s_{\mathrm{com}}\left(t\right)$ is known at each radar receiver after a previous estimation step [28]. In addition, it is supposed that the paths are stationary over the observation period. The communication BS carries out its task of information transmission by broadcasting signals throughout the space. Moreover, the radar antenna is directional and steered towards the target, and an adaptive beamforming technique is employed to reject interferences from other angles [15].

**Remark** **1** (Interference Mitigation)**.** *Some other techniques can be utilized to mitigate harmful interference for ultra-wide band systems, such as direct sequence (DS) and time-hopping sequence design [32], binary, quaternary and polyphase DS design [33], and blind selection of observations [33]. In addition, the interference can also be rejected by the type of radar waveform that is transmitted [34,35], which is not discussed due to the fact that it is out of the scope of this work.*

### 2.2. Signal Model

**Figure 2:**
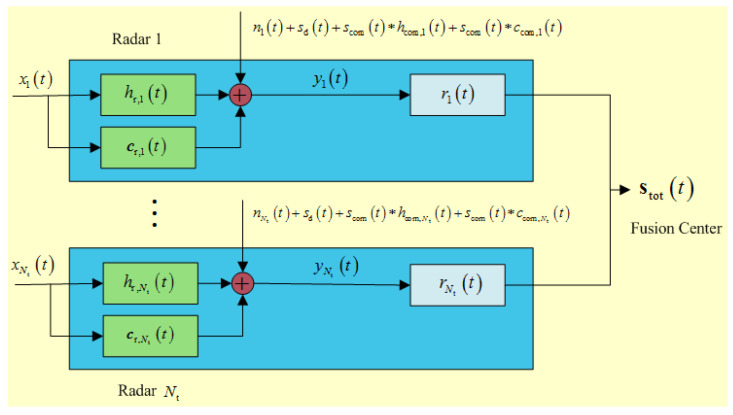
Signal model for radar waveform design in DMRS.

Figure 2 illustrates the known target signal model for multiple-radar waveform design. Let $s_{\mathrm{com}}\left(t\right)$ denote the continuous time-domain representation of a communication signal and $n_{i}\left(t\right)$ be the additive white Gaussian noise (AWGN) out of the radar receiver with PSD $S_{\mathrm{nn},i}\left(f\right)$, which is added on to the received signal of radar *i*. Unlike the radar’s signal that may be pulsed or continuous wave (CW), we assume that the communication signal is continuous during radar reception. $x_{i}\left(t\right)$ is the *i*-th complex-valued transmit waveform with finite duration $T_{i}$. $r_{i}\left(t\right)$ denotes the *i*-th complex-valued receiver filter impulse response, and $s_{\mathrm{tot}}\left(t\right)$ denotes the overall output signal, which can be mathematically expressed by $s_{\mathrm{tot}}\left(t\right)=\sum_{i=1}^{N_{t}}y_{i}\left(t\right)*r_{i}\left(t\right)$. In practice, when the radar return is received after waveform transmission, the signal-dependent clutter is also picked up and returned. Thus, the received signals at radar *i*’s receiver is given by:

(1) $$\begin{array}{c} y_{i}\left(t\right)=\underset{\mathrm{Target}\;\mathrm{return}}{\underset{︸}{x_{i}\left(t\right)*h_{r,i}\left(t\right)}}+\underset{\mathrm{Clutter}\;\mathrm{plus}\;\mathrm{interference}\;\mathrm{return}}{\underset{︸}{x_{i}\left(t\right)*c_{r,i}\left(t\right)+\underset{\mathrm{Communication}\;\mathrm{signals}}{\underset{︸}{\left[s_{d}\left(t\right)+s_{\mathrm{com}}\left(t\right)*h_{\mathrm{com}}\left(t\right)+s_{\mathrm{com}}\left(t\right)*c_{\mathrm{com},i}\left(t\right)\right]}}+n_{i}\left(t\right)}}, \end{array}$$

where $h_{r,i}\left(t\right)$ represents the target impulse response with respect to the *i*-th radar with finite duration $T_{h_{i}}$, $h_{\mathrm{com}}\left(t\right)$ represents the target impulse response with respect to the communication BS, $c_{r,i}\left(t\right)$ is the clutter response with respect to the *i*-th radar, and $c_{\mathrm{com},i}\left(t\right)$ is the clutter response with respect to the communication BS. Since the clutters $c_{r,i}\left(t\right)$ and $c_{\mathrm{com},i}\left(t\right)$ are usually random, we let $S_{\mathrm{ccr},i}\left(f\right)$ and $S_{\mathrm{ccs},i}\left(f\right)$ denote the PSDs of the corresponding clutter responses, which can be formed by the radar receiver through previous received signals before the target appears. In the frequency domain, the received signal can be expressed by [23]:

(2) $$\begin{array}{c} Y_{i}\left(f\right)=\underset{\mathrm{Target}\;\mathrm{return}}{\underset{︸}{|X_{i}{\left(f\right)|}^{2}{\left|H_{r,i}\left(f\right)\right|}^{2}L_{r,i}}}\\ +\underset{\mathrm{Clutter}\;\mathrm{plus}\;\mathrm{interference}\;\mathrm{return}}{\underset{︸}{|X_{i}{\left(f\right)|}^{2}S_{\mathrm{ccr},i}\left(f\right)L_{r,i}+\underset{\mathrm{Communication}\;\mathrm{signals}}{\underset{︸}{\left[|C_{\mathrm{com}}{\left(f\right)|}^{2}L_{d,i}+|C_{\mathrm{com}}{\left(f\right)|}^{2}|H_{\mathrm{com}}{\left(f\right)|}^{2}L_{s,i}+{\left|C_{\mathrm{com}}\left(f\right)\right|}^{2}S_{\mathrm{ccs},i}\left(f\right)L_{s,i}\right]}}+S_{\mathrm{nn},i}\left(f\right)}}, \end{array}$$

where $|X_{i}{\left(f\right)|}^{2}$ represents the energy spectral density (ESD) of the radar waveform, $L_{r,i}$ denotes the propagation loss of radar *i*-target-radar *i* path, $L_{d,i}$ denotes the propagation loss of the direct BS-radar *i* path, and $L_{s,i}$ denotes the propagation loss of BS-target-radar *i* path, which are given by [14]:

(3) $$\left\{\begin{array}{cc} L_{r,i} & =\begin{array}{c} \frac{G_{t,i}G_{r,i}\lambda_{i}^{2}}{{\left(4\pi \right)}^{3}d_{r,i}^{4}} \end{array},\\ L_{s,i} & =\begin{array}{c} \frac{G_{s}G_{r,i}\lambda_{i}^{2}}{{\left(4\pi \right)}^{3}d_{r,i}^{2}d_{s}^{2}} \end{array},\\ L_{d,i} & =\begin{array}{c} \frac{G_{s}G_{r,i}^{\prime }\lambda_{i}^{2}}{{\left(4\pi \right)}^{2}d_{i}^{2}} \end{array}, \end{array}\right.$$

where $G_{t,i}$ is the main-lobe transmitting antenna gain of radar *i*, $G_{r,i}$ is the main-lobe receiving antenna gain of radar *i*, $G_{r,i}^{{}^{\prime }}$ is the side-lobe receiving antenna gain of radar *i*, $G_{s}$ is the transmit/receive antenna gain of the communication BS, and $\lambda_{i}$ is the wavelength of radar *i*. We let $d_{r,i}$, $d_{s}$, and $d_{i}$ denote the distances between radar *i* and the target, between the communication BS and the target, and between the radar and the communication BS, respectively. In real application, the precise knowledge of propagation losses may be unavailable due to shot noise at radar receivers or atmospheric attenuation. One feasible method is to employ estimated values of these parameters in the optimal radar waveform optimization schemes. We can simplify Equation (2) by letting the clutter plus interference PSD be:

(4) $$\begin{array}{c} P_{i}\left(f\right)=\left[|C_{\mathrm{com}}{\left(f\right)|}^{2}L_{d,i}+|C_{\mathrm{com}}{\left(f\right)|}^{2}|H_{\mathrm{com}}{\left(f\right)|}^{2}L_{s,i}+{\left|C_{\mathrm{com}}\left(f\right)\right|}^{2}S_{\mathrm{ccs},i}\left(f\right)L_{s,i}\right]+S_{\mathrm{nn},i}\left(f\right), \end{array}$$

and thus Equation (2) can be written as:

(5) $$\begin{array}{c} Y_{i}\left(f\right)=|X_{i}{\left(f\right)|}^{2}|H_{r,i}{\left(f\right)|}^{2}L_{r,i}+{\left|X_{i}\left(f\right)\right|}^{2}S_{\mathrm{ccr},i}\left(f\right)L_{r,i}+P_{i}\left(f\right). \end{array}$$

As previously stated, all the path propagation gains are assumed to be fixed during observation.

## 3. Problem Formulation

### 3.1. Basis of the Technique

Mathematically, the LPI-based radar waveform design strategies for spectral coexistence of multiple-radar and communication systems in clutter can be described as a problem of optimizing the transmission waveform of each radar to minimize the total transmitted energy subject to a predefined performance requirement. Firstly, the analytical expressions of SCNR and MI are derived, where the communication signals received at the radar receiver are considered as interference. We are then in a position to optimize the optimal transmission waveform for DMRS coexisting with a communication system in order to achieve better LPI performance. The general LPI-based radar waveform design strategies are detailed as follows.

### 3.2. SCNR-Based Optimal Radar Waveform Design Strategy

As implied in [29,35], the SCNR is used as a metric for target detection performance in DMRS. It is assumed that the radar transmission waveform is essentially limited by its own bandwidth W. Based on the derivations in [22,29], the achievable SCNR can be described as:

(6) $$\begin{array}{c} \mathrm{SCNR}≜\sum_{i=1}^{N_{t}}\int_{-W/2}^{W/2}\frac{|X_{i}{\left(f\right)|}^{2}{\left|H_{r,i}\left(f\right)\right|}^{2}L_{r,i}}{|X_{i}{\left(f\right)|}^{2}S_{\mathrm{ccr},i}\left(f\right)L_{r,i}+P_{i}\left(f\right)}df. \end{array}$$

It can be noticed from Equation (6) that the achievable SCNR is related to the radar transmission waveform, the target spectra, the PSD of communication signal, the PSDs of the signal-dependent clutters, and the propagation losses of corresponding paths. Intuitively, maximization of SCNR means better target detection performance. However, it leads to transmitting much more energy, which in turn induces higher interference to the friendly communication system and increases the vulnerability of DMRS in modern battlefield.

In this paper, we concentrate on the LPI-based radar waveform design for the coexisting multiple-radar and wireless communication systems, whose objective is to minimize the total transmitted energy for a specified target performance such that the LPI performance is met. Eventually, the SCNR-based optimal radar waveform optimization can be formulated as:

(7) $$\begin{array}{cc} P_{\mathrm{SCNR}}:\;\;\; & \min_{|X_{i}{\left(f\right)|}^{2},f\in W}\;\;\sum_{i=1}^{N_{t}}\int_{-W/2}^{W/2}{\left|X_{i}\left(f\right)\right|}^{2}df,\\ & s.t.:\;\left\{\begin{array}{c} \sum_{i=1}^{N_{t}}\int_{-W/2}^{W/2}\frac{|X_{i}{\left(f\right)|}^{2}{\left|H_{r,i}\left(f\right)\right|}^{2}L_{r,i}}{|X_{i}{\left(f\right)|}^{2}S_{\mathrm{ccr},i}\left(f\right)L_{r,i}+P_{i}\left(f\right)}df\geq \gamma_{\mathrm{SCNR}},\\ \int_{-W/2}^{W/2}{\left|X_{i}\left(f\right)\right|}^{2}df\geq 0,\mathrm{for}\;\forall i. \end{array}\right. \end{array}$$

The first constraint stands that the achieved SCNR is greater than a predetermined SCNR threshold $\gamma_{\mathrm{SCNR}}$ such that the required target detection performance is met, while the second one represents that the transmit ESD of radar waveform is limited by a minimum value 0.

**Theorem** **1.** *Assuming perfect knowledge of the target spectra, the PSDs of clutter and communication signal, and the propagation losses of corresponding paths is available. Then, subject to a predefined SCNR threshold $\gamma_{\mathrm{SCNR}}$ and a transmit energy constraint, the optimal radar waveform corresponding to $P_{\mathrm{SCNR}}$ that minimizes the total transmitted energy should satisfy:*

(8) $$\begin{array}{c} |X_{i}^{*}{\left(f\right)|}^{2}=\max\left[0,B_{i}\left(f\right)\left(A^{*}-D_{i}\left(f\right)\right)\right],\;\;\mathrm{for}\;\forall i, \end{array}$$

*where*

(9) $$\left\{\begin{array}{cc} B_{i}\left(f\right) & =\frac{\sqrt{|H_{r,i}{\left(f\right)|}^{2}L_{r,i}P_{i}\left(f\right)}}{S_{\mathrm{ccr},i}\left(f\right)L_{r,i}},\\ D_{i}\left(f\right) & =\sqrt{\frac{P_{i}\left(f\right)}{|H_{r,i}{\left(f\right)|}^{2}L_{r,i}}}, \end{array}\right.$$

*and $A^{*}$ is a constant determined by:*

(10) $$\begin{array}{c} \sum_{i=1}^{N_{t}}\int_{-W/2}^{W/2}\frac{|X_{i}^{*}{\left(f\right)|}^{2}{\left|H_{r,i}\left(f\right)\right|}^{2}L_{r,i}}{|X_{i}^{*}{\left(f\right)|}^{2}S_{\mathrm{ccr},i}\left(f\right)L_{r,i}+P_{i}\left(f\right)}df\geq \gamma_{\mathrm{SCNR}}. \end{array}$$

**Proof** **of** **Theorem** **1.** Herein, to derive the closed-form solution, we employ the method of Lagrange multipliers to solve the constrained optimization problem (7). Introducing Lagrange multipliers $\xi_{i}\leq 0$ and μ≤0 for the multiple constraints, the Lagrange of problem $P_{\mathrm{SCNR}}$ can be equivalently expressed by:

(11) $$\begin{array}{cc} L(|X_{i}\left(f\right){|}^{2},\mu ,\xi_{i}) & =\sum_{i=1}^{N_{t}}\int_{-W/2}^{W/2}{\left|X_{i}\left(f\right)\right|}^{2}df+\mu \cdot \left(\sum_{i=1}^{N_{t}}\int_{-W/2}^{W/2}\frac{|X_{i}{\left(f\right)|}^{2}{\left|H_{r,i}\left(f\right)\right|}^{2}L_{r,i}}{|X_{i}{\left(f\right)|}^{2}S_{\mathrm{ccr},i}\left(f\right)L_{r,i}+P_{i}\left(f\right)}df-\gamma_{\mathrm{SCNR}}\right)\\ & -\sum_{i=1}^{N_{t}}\xi_{i}\cdot \int_{-W/2}^{W/2}{\left|X_{i}\left(f\right)\right|}^{2}df. \end{array}$$

This is equivalent to maximizing $l(|X_{i}\left(f\right){|}^{2},\mu ,\xi_{i})$ with respect to $|X_{i}{\left(f\right)|}^{2},$ where $l(|X_{i}\left(f\right){|}^{2},\mu ,\xi_{i})$ is given by:

(12) $$\begin{array}{cc} l(|X_{i}\left(f\right){|}^{2},\mu ,\xi_{i}) & =\frac{\partial L}{\partial |X_{i}{\left(f\right)|}^{2}}\\ & =\sum_{i=1}^{N_{t}}|X_{i}{\left(f\right)|}^{2}+\mu \cdot \sum_{i=1}^{N_{t}}\frac{|X_{i}{\left(f\right)|}^{2}{\left|H_{r,i}\left(f\right)\right|}^{2}L_{r,i}}{|X_{i}{\left(f\right)|}^{2}S_{\mathrm{ccr},i}\left(f\right)L_{r,i}+P_{i}\left(f\right)}-\sum_{i=1}^{N_{t}}\xi_{i}\cdot {\left|X_{i}\left(f\right)\right|}^{2}. \end{array}$$

In order to solve the problem $P_{\mathrm{SCNR}}$, the Karush–Kuhn–Tucker (KKT) optimality conditions can be subsequently derived as follows for any optimal point $\left(|X_{i}^{*}\left(f\right){|}^{2},\mu^{*},\xi_{i}^{*}\right)$:

(13) $$\left\{\begin{array}{c} \frac{\partial }{\partial |X_{i}^{*}{\left(f\right)|}^{2}}l(|X_{i}^{*}\left(f\right){|}^{2},\mu ,\xi_{i}){|}_{|X_{i}^{*}{\left(f\right)|}^{2},\mu^{*},\xi_{i}^{*}}=0,\\ \mu^{*}<0,\mathrm{if}\sum_{i=1}^{N_{t}}\int_{-W/2}^{W/2}\frac{|X_{i}^{*}{\left(f\right)|}^{2}{\left|H_{r,i}\left(f\right)\right|}^{2}L_{r,i}}{|X_{i}^{*}{\left(f\right)|}^{2}S_{\mathrm{ccr},i}\left(f\right)L_{r,i}+P_{i}\left(f\right)}df=\gamma_{\mathrm{SCNR}},\\ \mu^{*}=0,\mathrm{if}\sum_{i=1}^{N_{t}}\int_{-W/2}^{W/2}\frac{|X_{i}^{*}{\left(f\right)|}^{2}{\left|H_{r,i}\left(f\right)\right|}^{2}L_{r,i}}{|X_{i}^{*}{\left(f\right)|}^{2}S_{\mathrm{ccr},i}\left(f\right)L_{r,i}+P_{i}\left(f\right)}df>\gamma_{\mathrm{SCNR}},\\ \xi_{i}^{*}<0,\mathrm{if}\int_{-W/2}^{W/2}{\left|X_{i}^{*}\left(f\right)\right|}^{2}df=0,\\ \xi_{i}^{*}=0,\mathrm{if}\int_{-W/2}^{W/2}{\left|X_{i}^{*}\left(f\right)\right|}^{2}df>0,\\ \xi_{i}^{*}\leq 0,\\ \mu \leq 0. \end{array}\right.$$

From the stationary condition, when $|X_{i}^{*}{\left(f\right)|}^{2}$ is optimal, we obtain:

(14) $$\begin{array}{c} |X_{i}^{*}{\left(f\right)|}^{2}=-\frac{P_{i}\left(f\right)}{S_{\mathrm{ccr},i}\left(f\right)L_{r,i}}\pm \frac{\sqrt{\left(-\mu \right)P_{i}\left(f\right){\left|H_{r,i}\left(f\right)\right|}^{2}L_{r,i}}}{S_{\mathrm{ccr},i}\left(f\right)L_{r,i}}. \end{array}$$

Setting $A=\sqrt{-\mu }$, rearranging terms, and ensuring $|X_{i}^{*}{\left(f\right)|}^{2}$ to be positive, the $|X_{i}^{*}{\left(f\right)|}^{2}$ that minimizes $\sum_{i=1}^{N_{t}}\int_{-W/2}^{W/2}{\left|X_{i}\left(f\right)\right|}^{2}$ is given by:

(15) $$\begin{array}{c} |X_{i}^{*}{\left(f\right)|}^{2}=\max\left[0,\frac{\sqrt{|H_{r,i}{\left(f\right)|}^{2}L_{r,i}P_{i}\left(f\right)}}{S_{\mathrm{ccr},i}\left(f\right)L_{r,i}}\left(A^{*}-\sqrt{\frac{P_{i}\left(f\right)}{|H_{r,i}{\left(f\right)|}^{2}L_{r,i}}}\right)\right],\;\;\mathrm{for}\;\forall i, \end{array}$$

where $A^{*}$ is called *water-level* determined by:

(16) $$\begin{array}{c} \sum_{i=1}^{N_{t}}\int_{-W/2}^{W/2}\frac{|X_{i}^{*}{\left(f\right)|}^{2}{\left|H_{r,i}\left(f\right)\right|}^{2}L_{r,i}}{|X_{i}^{ite}{\left(f\right)|}^{2}S_{\mathrm{ccr},i}\left(f\right)L_{r,i}+P_{i}\left(f\right)}df\geq \gamma_{\mathrm{SCNR}}. \end{array}$$

Therefore, the SCNR-based optimal radar waveform can be derived as Equation (8), which completes the proof. ☐

**Remark** **2** (Algorithm Analysis)**.** *We can notice from Equation (8) that the SCNR-based radar waveform design is well-known to be a "water-filling" solution by minimizing the total transmitted energy. The bisection search technique is exploited to actually find the optimal value of $A^{*}$ that ensures the optimum radar waveform $|X_{i}^{*}{\left(f\right)|}^{2}$ while making sure that both constraints are satisfied. The importance of the derived solution (8) lies in the fact that it provides an explicit relation between the transmitted radar waveform over the whole frequency bands and the resulting value of $A^{*}$. Problem $P_{\mathrm{SCNR}}$ defines a procedure that finally provides the optimal radar transmission waveform, and, consequently, the optimum LPI performance. The iterative procedure to solve the problem of optimal radar waveform design is detailed in Algorithm 1. The bisection search algorithm is listed as Algorithm 2.*

**Algorithm 1** Optimal Radar Waveform Design for $P_{\mathrm{SCNR}}$
1:**Initialization:** $\gamma_{\mathrm{SCNR}}$, iterative index ite=1; 2:**Loop until** $|X_{i}^{\left(ite\right)}{\left(f\right)|}^{2}$ **converges:**
  **for** *i* = 1, ⋯, $N_{t}$, **do**
   Calculate $|X_{i}^{\left(ite\right)}{\left(f\right)|}^{2}$ by solving (8);   Calculate ${\mathrm{SCNR}}^{\left(ite\right)}\leftarrow \sum_{i=1}^{N_{t}}\int_{-W/2}^{W/2}\frac{|X_{i}^{ite}{\left(f\right)|}^{2}{\left|H_{r,i}\left(f\right)\right|}^{2}L_{r,i}}{|X_{i}^{ite}{\left(f\right)|}^{2}S_{\mathrm{ccr},i}\left(f\right)L_{r,i}+P_{i}\left(f\right)}df$;   Obtain $A^{\left(ite+1\right)}$ via bisection search in Algorithm 2;   **end for**3:**End loop**
4:**Update:** Update $|X_{i}^{*}{\left(f\right)|}^{2}\leftarrow {\left|X_{i}^{ite}\left(f\right)\right|}^{2}$ for ∀i.


**Algorithm 2** Bisection Search of *A*
1:**Initialization:** $A^{\left(ite\right)}$, $A_{\max}$, $A_{\min}$, the tolerance ε>0; 2:**Loop until:** ${\mathrm{SCNR}}^{\left(ite\right)}-\gamma_{\mathrm{SCNR}}\geq \varepsilon$ **for** *i* = 1, ⋯, $N_{t}$, **do**
   $A^{\left(ite\right)}\leftarrow \left(A_{\min}+A_{\max}\right)/2$;    Calculate $|X_{i}^{ite}{\left(f\right)|}^{2}$ from (8) and update ${\mathrm{SCNR}}^{\left(ite\right)}$;    **if** ${\mathrm{SCNR}}^{\left(ite\right)}>\gamma_{\mathrm{SCNR}}$**then**
    $A_{\max}\leftarrow A^{\left(ite\right)}$;    **else**
    $A_{\min}\leftarrow A^{\left(ite\right)}$;    **end if**
   $A^{\left(ite\right)}\leftarrow \left(A_{\min}+A_{\max}\right)/2$;    Set ite←ite+1;   **end for**
3:**End loop**

**Remark** **3** (Bound for *A*)**.** *It is noteworthy that Equation (8) is only valid within certain conditions [23]. Firstly, from the numerator of the non-zero term in Equation (14), one can observe that:*

(17) $$\begin{array}{c} A\cdot \sqrt{P_{i}\left(f\right){\left|H_{r,i}\left(f\right)\right|}^{2}L_{r,i}}>P_{i}\left(f\right) \end{array}$$

*for the output of Equation (8) to remain positive; otherwise, Equation (8) leads to zero. Dividing the term $\sqrt{P_{i}\left(f\right){\left|H_{r,i}\left(f\right)\right|}^{2}L_{r,i}}$, we can obtain:*

(18) $$\begin{array}{c} A>\sqrt{\frac{P_{i}\left(f\right)}{|H_{r,i}{\left(f\right)|}^{2}L_{r,i}}}. \end{array}$$

Now, we will calculate the upper bound for *A*, which is much more complicated. For the solution to be valid and to water-fill the frequency band due to $P_{i}\left(f\right)$, the non-zero term of Equation (14) can be regarded as a function of $P_{i}\left(f\right)$. Then, the first derivative of the function with respect to $P_{i}\left(f\right)$ can be expressed by:

(19) $$\begin{array}{c} \frac{\partial |X_{i}{\left(f\right)|}^{2}}{\partial P_{i}\left(f\right)}=-\frac{1}{S_{\mathrm{ccr},i}\left(f\right)L_{r,i}}+\frac{A|H_{r,i}\left(f\right)|}{2\sqrt{P_{i}\left(f\right)L_{r,i}}S_{\mathrm{ccr},i}\left(f\right)}. \end{array}$$

For fixed values of *A* and $P_{i}\left(f\right)$, as $P_{i}\left(f\right)$ approaches zero, $\frac{\partial |X_{i}{\left(f\right)|}^{2}}{\partial P_{i}\left(f\right)}$ is mostly positive and approaches infinity. Due to the fact that Equation (8) needs to be applied in portions where its output values are decreasing, the variable *A* must be such that the first derivative remains negative throughout the range of $P_{i}\left(f\right)$. Thus, setting $\frac{\partial |X_{i}{\left(f\right)|}^{2}}{\partial P_{i}\left(f\right)}$ less than zero, we can obtain:

(20) $$\begin{array}{c} \frac{A|H_{r,i}\left(f\right)|}{2\sqrt{P_{i}\left(f\right)L_{r,i}}S_{\mathrm{ccr},i}\left(f\right)}<\frac{1}{S_{\mathrm{ccr},i}\left(f\right)L_{r,i}}, \end{array}$$

which can be simplified to the following result:

(21) $$\begin{array}{c} A<2\sqrt{\frac{P_{i}\left(f\right)}{|H_{r,i}{\left(f\right)|}^{2}L_{r,i}}}. \end{array}$$

Hence, the bounds of the water-filling variable *A* are determined to be:

(22) $$\begin{array}{c} \sqrt{\frac{P_{i}\left(f\right)}{|H_{r,i}{\left(f\right)|}^{2}L_{r,i}}}<A<2\sqrt{\frac{P_{i}\left(f\right)}{|H_{r,i}{\left(f\right)|}^{2}L_{r,i}}}. \end{array}$$

It is obvious from Equation (22) that no energy will be filled in $|X_{i}{\left(f\right)|}^{2}$ if *A* is chosen to be below $\sqrt{\frac{P_{i}\left(f\right)}{|H_{r,i}{\left(f\right)|}^{2}L_{r,i}}}$ or above $2\sqrt{\frac{P_{i}\left(f\right)}{|H_{r,i}{\left(f\right)|}^{2}L_{r,i}}}$.

### 3.3. MI-Based Optimal Radar Waveform Design Strategy

Next, we utilize the MI between the received echo and the target impulse response as a metric for target characterization performance in DMRS. The achievable MI can be expressed by:

(23) $$\begin{array}{c} \mathrm{MI}≜\sum_{i=1}^{N_{t}}T_{y_{i}}\cdot \int_{-W/2}^{W/2}\ln\left(1+\frac{|X_{i}{\left(f\right)|}^{2}{\left|H_{r,i}\left(f\right)\right|}^{2}L_{r,i}}{T_{y_{i}}\left[|X_{i}{\left(f\right)|}^{2}S_{\mathrm{ccr},i}\left(f\right)L_{r,i}+P_{i}\left(f\right)\right]}\right)df, \end{array}$$

where $T_{y_{i}}=T_{i}+T_{h_{i}}$ denotes the duration of the target return $y_{i}\left(t\right)$. For simplicity, it is assumed that $T_{y_{i}}=T_{y}$ for ∀i. Then, Equation (23) can be simplified as follows:

(24) $$\begin{array}{c} \mathrm{MI}≜\sum_{i=1}^{N_{t}}T_{y}\cdot \int_{-W/2}^{W/2}\ln\left(1+\frac{|X_{i}{\left(f\right)|}^{2}{\left|H_{r,i}\left(f\right)\right|}^{2}L_{r,i}}{T_{y}\left[|X_{i}{\left(f\right)|}^{2}S_{\mathrm{ccr},i}\left(f\right)L_{r,i}+P_{i}\left(f\right)\right]}\right)df. \end{array}$$

From Equation (24), we can see that the achievable MI is related to the radar transmission waveform, the target spectra, the PSD of communication signal, the PSDs of the signal-dependent clutters, and the propagation losses of corresponding paths. Intuitively, maximization of MI means better target estimation performance, which also results in worse LPI performance. Similarly, the MI-based optimal radar waveform design strategy is developed as follows:

(25) $$\begin{array}{cc} P_{\mathrm{MI}}:\;\;\; & \min_{|X_{i}{\left(f\right)|}^{2},f\in W}\;\;\sum_{i=1}^{N_{t}}\int_{-W/2}^{W/2}{\left|X_{i}\left(f\right)\right|}^{2}df,\\ & s.t.:\;\left\{\begin{array}{c} \sum_{i=1}^{N_{t}}T_{y}\cdot \int_{-W/2}^{W/2}\ln\left(1+\frac{|X_{i}{\left(f\right)|}^{2}{\left|H_{r,i}\left(f\right)\right|}^{2}L_{r,i}}{T_{y}\left[|X_{i}{\left(f\right)|}^{2}S_{\mathrm{ccr},i}\left(f\right)L_{r,i}+P_{i}\left(f\right)\right]}\right)df\geq \gamma_{\mathrm{MI}},\\ \int_{-W/2}^{W/2}{\left|X_{i}\left(f\right)\right|}^{2}df\geq 0,\mathrm{for}\;\forall i, \end{array}\right. \end{array}$$

where $\gamma_{\mathrm{MI}}$ denotes the predefined MI threshold.

**Theorem** **2.** *Assuming perfect knowledge of the target spectra, the PSDs of clutter and communication signal, and the propagation losses of corresponding paths are available. Then, subject to a predefined MI threshold $\gamma_{\mathrm{MI}}$ and a transmit energy constraint, the optimal radar waveform corresponding to $P_{\mathrm{MI}}$ that minimizes the total transmitted energy should satisfy:*

(26) $$\begin{array}{c} |X_{i}^{*}{\left(f\right)|}^{2}\approx \max\left[0,B_{i}\left(f\right)\left(A^{*}-D_{i}\left(f\right)\right)\right],\;\;\mathrm{for}\;\forall i, \end{array}$$

*where*

(27) $$\left\{\begin{array}{cc} B_{i}\left(f\right) & =\frac{|H_{r,i}{\left(f\right)|}^{2}/T_{y}}{2S_{\mathrm{ccr},i}\left(f\right)+{\left|H_{r,i}\left(f\right)\right|}^{2}/T_{y}},\\ D_{i}\left(f\right) & =\frac{P_{i}\left(f\right)}{|H_{r,i}{\left(f\right)|}^{2}L_{r,i}/T_{y}}, \end{array}\right.$$

*and $A^{*}$ is a constant determined by:*

(28) $$\begin{array}{c} \sum_{i=1}^{N_{t}}T_{y}\cdot \int_{-W/2}^{W/2}ln\left(1+\frac{|X_{i}^{*}{\left(f\right)|}^{2}{\left|H_{r,i}\left(f\right)\right|}^{2}L_{r,i}}{T_{y}\left[|X_{i}^{*}{\left(f\right)|}^{2}S_{\mathrm{ccr},i}\left(f\right)L_{r,i}+P_{i}\left(f\right)\right]}\right)df\geq \gamma_{\mathrm{MI}}. \end{array}$$

**Proof** **of** **Theorem** **2.** Similarly, we invoke the Lagrange multiplier technique yielding the following objective function:

(29) $$\begin{array}{cc} L(|X_{i}\left(f\right){|}^{2},\mu ,\xi_{i}) & =\sum_{i=1}^{N_{t}}\int_{-W/2}^{W/2}{\left|X_{i}\left(f\right)\right|}^{2}df+\mu \cdot \left[\sum_{i=1}^{N_{t}}T_{y}\cdot \int_{-W/2}^{W/2}\ln\left(1+\frac{|X_{i}{\left(f\right)|}^{2}{\left|H_{r,i}\left(f\right)\right|}^{2}L_{r,i}}{T_{y}\left[|X_{i}{\left(f\right)|}^{2}S_{\mathrm{ccr},i}\left(f\right)L_{r,i}+P_{i}\left(f\right)\right]}\right)df-\gamma_{\mathrm{MI}}\right]\\ & -\sum_{i=1}^{N_{t}}\xi_{i}\cdot \int_{-W/2}^{W/2}{\left|X_{i}\left(f\right)\right|}^{2}df. \end{array}$$

This is equivalent to maximizing $l(|X_{i}\left(f\right){|}^{2},\mu ,\xi_{i})$ with respect to $|X_{i}{\left(f\right)|}^{2}$ where $l(|X_{i}\left(f\right){|}^{2},\mu ,\xi_{i})$ is given by:

(30) $$\begin{array}{cc} l(|X_{i}\left(f\right){|}^{2},\mu ,\xi_{i}) & =\frac{\partial L}{\partial |X_{i}{\left(f\right)|}^{2}}\\ & =\sum_{i=1}^{N_{t}}|X_{i}{\left(f\right)|}^{2}+\mu \cdot \sum_{i=1}^{N_{t}}T_{y}\cdot \ln\left(1+\frac{|X_{i}{\left(f\right)|}^{2}{\left|H_{r,i}\left(f\right)\right|}^{2}L_{r,i}}{T_{y}\left[|X_{i}{\left(f\right)|}^{2}S_{\mathrm{ccr},i}\left(f\right)L_{r,i}+P_{i}\left(f\right)\right]}\right)-\sum_{i=1}^{N_{t}}\xi_{i}\cdot {\left|X_{i}\left(f\right)\right|}^{2}. \end{array}$$

In order to solve the problem $P_{\mathrm{MI}}$, the KKT conditions can be developed as follows:

(31) $$\left\{\begin{array}{c} \frac{\partial }{\partial |X_{i}^{*}{\left(f\right)|}^{2}}l(|X_{i}^{*}\left(f\right){|}^{2},\mu ,\xi_{i}){|}_{|X_{i}^{*}{\left(f\right)|}^{2},\mu^{*},\xi_{i}^{*}}=0,\\ \mu^{*}<0,\mathrm{if}\sum_{i=1}^{N_{t}}T_{y}\cdot \int_{-W/2}^{W/2}\ln\left(1+\frac{|X_{i}^{*}{\left(f\right)|}^{2}{\left|H_{r,i}\left(f\right)\right|}^{2}L_{r,i}}{T_{y}\left[|X_{i}^{*}{\left(f\right)|}^{2}S_{\mathrm{ccr},i}\left(f\right)L_{r,i}+P_{i}\left(f\right)\right]}\right)df=\gamma_{\mathrm{MI}},\\ \mu^{*}=0,\mathrm{if}\sum_{i=1}^{N_{t}}T_{y}\cdot \int_{-W/2}^{W/2}\ln\left(1+\frac{|X_{i}^{*}{\left(f\right)|}^{2}{\left|H_{r,i}\left(f\right)\right|}^{2}L_{r,i}}{T_{y}\left[|X_{i}^{*}{\left(f\right)|}^{2}S_{\mathrm{ccr},i}\left(f\right)L_{r,i}+P_{i}\left(f\right)\right]}\right)df>\gamma_{\mathrm{MI}},\\ \xi_{i}^{*}<0,\mathrm{if}\int_{-W/2}^{W/2}{\left|X_{i}^{*}\left(f\right)\right|}^{2}df=0,\\ \xi_{i}^{*}=0,\mathrm{if}\int_{-W/2}^{W/2}{\left|X_{i}^{*}\left(f\right)\right|}^{2}df>0,\\ \xi_{i}^{*}\leq 0,\\ \mu \leq 0. \end{array}\right.$$

From the stationary condition, when $|X_{i}^{*}{\left(f\right)|}^{2}$ is optimal, we obtain:

(32) $$\begin{array}{c} |X_{i}^{*}{\left(f\right)|}^{2}=\max\left[0,-R_{i}\left(f\right)+\sqrt{R_{i}^{2}\left(f\right)+S_{i}\left(f\right)\left(A-D_{i}\left(f\right)\right)}\right],\;\;\mathrm{for}\;\forall i, \end{array}$$

where

(33) $$\left\{\begin{array}{cc} R_{i}\left(f\right) & =\frac{P_{i}\left(f\right)(2S_{\mathrm{ccr},i}\left(f\right)+|H_{r,i}\left(f\right){|}^{2}/T_{y})}{2S_{\mathrm{ccr},i}\left(f\right)L_{r,i}(S_{\mathrm{ccr},i}\left(f\right)+|H_{r,i}\left(f\right){|}^{2}/T_{y})},\\ S_{i}\left(f\right) & =\frac{P_{i}\left(f\right){\left|H_{r,i}\left(f\right)\right|}^{2}/T_{y}}{S_{\mathrm{ccr},i}\left(f\right)L_{r,i}(S_{\mathrm{ccr},i}\left(f\right)+|H_{r,i}\left(f\right){|}^{2}/T_{y})},\\ D_{i}\left(f\right) & =\frac{P_{i}\left(f\right)}{|H_{r,i}{\left(f\right)|}^{2}L_{r,i}/T_{y}}, \end{array}\right.$$

and the constant $A=\left(-\mu \right)T_{y}$ is determined by the MI constraint:

(34) $$\begin{array}{c} \sum_{i=1}^{N_{t}}T_{y}\cdot \int_{-W/2}^{W/2}\ln\left(1+\frac{|X_{i}^{*}{\left(f\right)|}^{2}{\left|H_{r,i}\left(f\right)\right|}^{2}L_{r,i}}{T_{y}\left[|X_{i}^{*}{\left(f\right)|}^{2}S_{\mathrm{ccr},i}\left(f\right)L_{r,i}+P_{i}\left(f\right)\right]}\right)df\geq \gamma_{\mathrm{MI}}. \end{array}$$

To gain further intuition, we apply a first-order Taylor approximation to Equation (32), yielding:

(35) $$\begin{array}{cc} Q_{i}\left(f\right) & =-R_{i}\left(f\right)+\sqrt{R_{i}^{2}\left(f\right)+S_{i}\left(f\right)\left(A-D_{i}\left(f\right)\right)}\\ & \approx B_{i}\left(f\right)\left(A-D_{i}\left(f\right)\right), \end{array}$$

where

(36) $$\begin{array}{cc} B_{i}\left(f\right) & =\frac{|H_{r,i}{\left(f\right)|}^{2}/T_{y}}{2S_{\mathrm{ccr},i}\left(f\right)+{\left|H_{r,i}\left(f\right)\right|}^{2}/T_{y}}. \end{array}$$

Thus, the radar waveform is approximated by:

(37) $$\begin{array}{c} |X_{i}^{*}{\left(f\right)|}^{2}\approx \max\left[0,B_{i}\left(f\right)\left(A^{*}-D_{i}\left(f\right)\right)\right],\;\;\mathrm{for}\;\forall i. \end{array}$$

Therefore, the MI-based optimal radar waveform can be derived as Equation (8), which completes the proof. ☐

**Remark** **4** (Algorithm Analysis)**.** *The MI-based optimal radar waveform design strategy also performs “water-filling” operations, and A controls the desired MI threshold. The iterative procedure of problem $P_{\mathrm{MI}}$ is summarized in Algorithm 3.*

**Algorithm 3** Optimal Radar Waveform Design for $P_{\mathrm{MI}}$
1:**Initialization:** $\gamma_{\mathrm{MI}}$, iterative index ite=1; 2:**Loop until**$|X_{i}^{\left(ite\right)}{\left(f\right)|}^{2}$**converges:**
 **for** *i* = 1, ⋯, $N_{t}$, **do**
  Calculate $|X_{i}^{\left(ite\right)}{\left(f\right)|}^{2}$ by solving (26);  Calculate ${\mathrm{MI}}^{\left(ite\right)}\leftarrow \sum_{i=1}^{N_{t}}T_{y}\cdot \int_{-W/2}^{W/2}\ln\left(1+\frac{|X_{i}^{\left(ite\right)}{\left(f\right)|}^{2}{\left|H_{r,i}\left(f\right)\right|}^{2}L_{r,i}}{T_{y}\left[|X_{i}^{\left(ite\right)}{\left(f\right)|}^{2}S_{\mathrm{ccr},i}\left(f\right)L_{r,i}+P_{i}\left(f\right)\right]}\right)df$;   Obtain $A^{\left(ite+1\right)}$ via bisection search in Algorithm 2;  **end for**
3:**End loop**
4:**Update:** Update $|X_{i}^{*}{\left(f\right)|}^{2}\leftarrow {\left|X_{i}^{ite}\left(f\right)\right|}^{2}$ for ∀i.

**Remark** **5** (Bound for *A*)**.** *Note that Equation (26) is only valid within certain conditions. Similarly, for the output of Equation (26) to remain positive, we can obtain:*

(38) $$\begin{array}{c} B_{i}\left(f\right)\left(A-D_{i}\left(f\right)\right)>0; \end{array}$$

*thus, we have*

(39) $$\begin{array}{c} A>\frac{P_{i}\left(f\right)}{|H_{r,i}{\left(f\right)|}^{2}L_{r,i}/T_{y}}. \end{array}$$

Then, taking the first derivative of the function (26) with respect to $P_{i}\left(f\right)$ yields:

(40) $$\begin{array}{cc} \frac{\partial |X_{i}{\left(f\right)|}^{2}}{\partial P_{i}\left(f\right)} & =-\frac{|H_{r,i}{\left(f\right)|}^{2}/T_{y}}{2S_{\mathrm{ccr},i}\left(f\right)+{\left|H_{r,i}\left(f\right)\right|}^{2}/T_{y}}\cdot \frac{1}{|H_{r,i}{\left(f\right)|}^{2}L_{r,i}/T_{y}}\\ & =-\frac{1}{2S_{\mathrm{ccr},i}\left(f\right)L_{r,i}+{\left|H_{r,i}\left(f\right)\right|}^{2}L_{r,i}/T_{y}}<0. \end{array}$$

From Equation (39), we can observe that, for any value of *A*, the first derivative remains negative throughout the range of $P_{i}\left(f\right)$.

Therefore, the bound of the water-filling variable *A* is determined to be:

(41) $$\begin{array}{c} A>\frac{P_{i}\left(f\right)}{|H_{r,i}{\left(f\right)|}^{2}L_{r,i}/T_{y}}. \end{array}$$

It is apparent from Equation (40) that no energy will be filled in $|X_{i}{\left(f\right)|}^{2}$ if *A* is chosen to be below $\frac{P_{i}\left(f\right)}{|H_{r,i}{\left(f\right)|}^{2}L_{r,i}/T_{y}}$.

### 3.4. Potential Extension

Without loss of generality, we look into a single-target case in this work. However, the calculations and results can be extended to the multiple-target scenario, in which each radar transmitter can launch multiple beams simultaneously to execute different radar tasks. In this mode, each transmit beam can be used to detect or track one target, and thus multiple targets can be probed. For multiple-target cases, the resulting SCNR-based optimal radar waveform optimization can be reformulated as:

(42) $$\begin{array}{cc} P_{\mathrm{SCNR}}^{\prime }:\;\;\; & \min_{|X_{i}^{q}{\left(f\right)|}^{2},f\in W}\;\;\sum_{i=1}^{N_{t}}\sum_{q=1}^{Q}\int_{-W/2}^{W/2}{\left|X_{i}^{q}\left(f\right)\right|}^{2}df,\\ & s.t.:\;\left\{\begin{array}{c} \sum_{i=1}^{N_{t}}\int_{-W/2}^{W/2}\frac{|X_{i}^{q}{\left(f\right)|}^{2}{\left|H_{r,i}^{q}\left(f\right)\right|}^{2}L_{r,i}^{q}}{|X_{i}^{q}{\left(f\right)|}^{2}S_{\mathrm{ccr},i}^{q}\left(f\right)L_{r,i}^{q}+P_{i}^{q}\left(f\right)}df\geq \gamma_{\mathrm{SCNR}},\\ \int_{-W/2}^{W/2}{\left|X_{i}^{q}\left(f\right)\right|}^{2}df\geq 0,\mathrm{for}\;\forall i, \end{array}\right. \end{array}$$

where all the parameters with superscript *q* denote the corresponding ones of target *q*. Similarly, the MI-based optimal radar waveform optimization for multiple-target case can be developed as:

(43) $$\begin{array}{cc} P_{\mathrm{MI}}^{\prime }:\;\;\; & \min_{|X_{i}^{q}{\left(f\right)|}^{2},f\in W}\;\;\sum_{i=1}^{N_{t}}\sum_{q=1}^{Q}\int_{-W/2}^{W/2}{\left|X_{i}^{q}\left(f\right)\right|}^{2}df,\\ & s.t.:\;\left\{\begin{array}{c} \sum_{i=1}^{N_{t}}T_{y}^{q}\cdot \int_{-W/2}^{W/2}\ln\left(1+\frac{|X_{i}^{q}{\left(f\right)|}^{2}{\left|H_{r,i}^{q}\left(f\right)\right|}^{2}L_{r,i}^{q}}{T_{y}^{q}\left[|X_{i}^{q}{\left(f\right)|}^{2}S_{\mathrm{ccr},i}^{q}\left(f\right)L_{r,i}^{q}+P_{i}^{q}\left(f\right)\right]}\right)df\geq \gamma_{\mathrm{MI}},\\ \int_{-W/2}^{W/2}{\left|X_{i}^{q}\left(f\right)\right|}^{2}df\geq 0,\mathrm{for}\;\forall i. \end{array}\right. \end{array}$$

Then, we can also employ the bisection approach to search for the optimal waveform design results for $P_{\mathrm{SCNR}}^{\prime }$ and $P_{\mathrm{MI}}^{\prime }$. In this scenario, it can be concluded that the proposed radar waveform design schemes can easily be extended to multiple-target cases by adding the transmit energy for each target.

### 3.5. Discussion

(1) The LPI-based radar waveform design strategies are obtained when the target spectra, the PSD of communication signal, the PSDs of the signal-dependent clutters, and the propagation losses of corresponding paths are assumed to be perfectly known. The SCNR- and MI-based optimization criteria are chosen based on different radar tasks. By employing the designed waveforms, the transmitted energy of DMRS can be minimized and used most efficiently to achieve the best LPI performance.
(2) Note that since the designed optimal radar transmission waveforms are phase tolerant, there would be a number of time-domain waveforms that fit the spectrum [23].
(3) From Equations (6) and (24), it should be pointed out that MI is a function of SCNR. Since the calculation of MI involves the log computations, there will be less dominant frequency components in the MI-based radar waveform design strategy [36]. Moreover, more frequencies will be allocated energy via a water-filling operation. In the following, simulation results will illustrate that the proposed two radar waveform design strategies actually lead to different energy allocation results.
(4) This paper proposes the optimal radar waveform design strategies based on two different applications, that is, target detection and target characterization. The SCNR-based optimal radar waveform optimization strategy designs a waveform that maximizes the energy of the signals scattered off the target. In this scenario, we only focus on capturing the peak of the target spectrum to detect the target, and thus the extractable information about the target is much less. While the MI-based optimal radar waveform optimization strategy designs a spectrally efficient waveform with a wide bandwidth, which has a better range resolution than a traditional pulsed radar signal. In this case, much transmission energy is distributed over the whole frequency band, which is good for target characterization.
(5) This paper proposes the SCNR- and MI-based optimal radar waveform design schemes under the quite idealistic assumption of perfectly known target spectra, PSD of communication signal, PSDs of the signal-dependent clutters, and propagation losses of corresponding paths. However, the proposed radar waveform design schemes can be extended straightforwardly to the robust ones. In real application, the precise knowledge of the target spectra, PSD of communication signal, PSDs of the signal-dependent clutters, and propagation losses of the corresponding paths are usually not available. One feasible approach is to employ the uncertainty model, where these parameters are assumed to lie in uncertainty sets bounded by known upper and lower bounds. The corresponding robust radar waveform design schemes are omitted here due to space limitations. Detailed uncertainty model can refer to [22,28]. It is indicated in [22] that the robust waveforms can bound the worst-case LPI performance of the DMRS for any parameters in the uncertainty sets.
(6) Note that the proposed optimal radar waveform design schemes only present the optimal waveform amplitude in frequency-domain. The phase information of the transmitted signal can be determined by utilizing the cyclic iteration approach and minimum mean-square error (MMSE) criterion. The optimal radar waveform design for DMRS in time-domain will be investigated in future work.

## 4. Numerical Results and Performance Analysis

In this section, we provide numerical results to demonstrate the accuracy of the theoretical calculations as well as quantify the LPI performance of the proposed radar waveform design strategies for the coexistence of the distributed multiple-radar and wireless communication systems.

### 4.1. Numerical Setup

**Figure 3:**
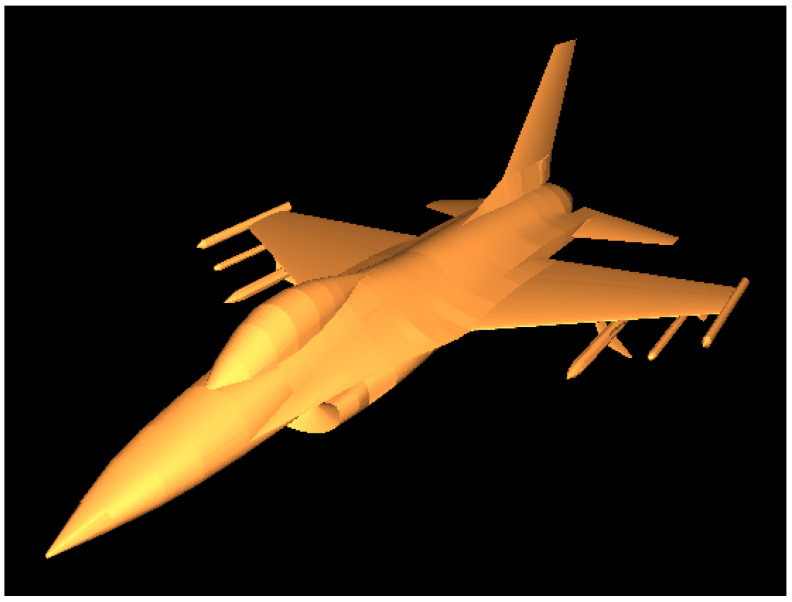
Simulated target model.

**Figure 4:**
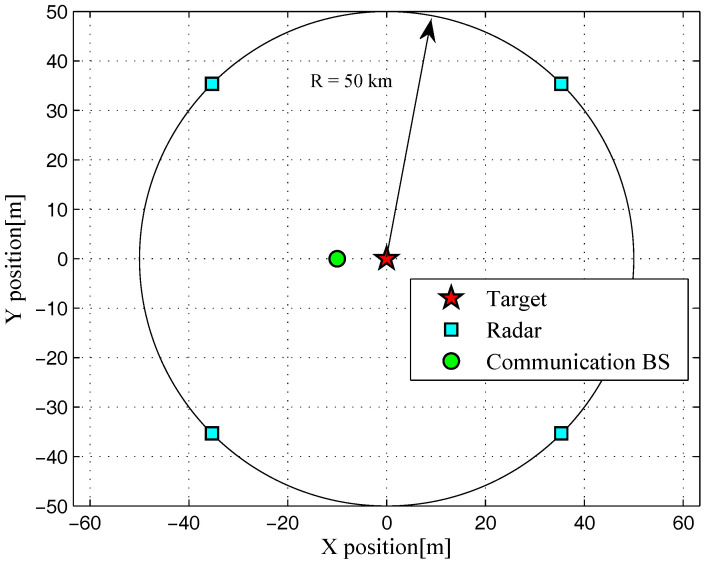
Simulated 2D scenario with locations of multiple radars, communication BS and target.

Throughout the numerical simulations, it is assumed a DMRS with $N_{t}=4$ radars. The simulated target model is shown in Figure 3, which can precisely be modeled through the geometry modeling of complex target, such as the grid model used in the faceting approach, the parametric surface approach and the decompounding approach. The locations of multiple radars, communication BS and target are illustrated in Figure 4. The carrier frequency of the coexisting multiple-radar and communication systems is 3 GHz. Here, DMRS can access the whole frequency band with a desired target detection performance constraint $\gamma_{\mathrm{SCNR}}=9\;\mathrm{dB}$, which is approximately equivalent to the value of $\gamma_{\mathrm{MI}}=2.85\;\mathrm{dB}$ for a specified target characterization requirement. Unless otherwise stated, we utilize the default values for the system parameters as given in Table 1. To solve the problems of optimal radar waveform design $P_{\mathrm{SCNR}}$ and $P_{\mathrm{MI}}$, it is assumed that DMRS knows the exact characteristics of the target spectra, the PSD of communication signal, the PSDs of the signal-dependent clutters, and the propagation losses of corresponding paths by sensing itself with a spectrum analyzer.

### 4.2. Radar Waveform Design Results

Define the target-to-interference-plus-noise ratio (TINR) of the *i*-th radar as:

(44) $$\begin{array}{c} {\mathrm{TINR}}_{i}\left(f\right)≜\frac{|H_{r,i}{\left(f\right)|}^{2}L_{r,i}}{P_{i}\left(f\right)}. \end{array}$$

**Figure 5:**
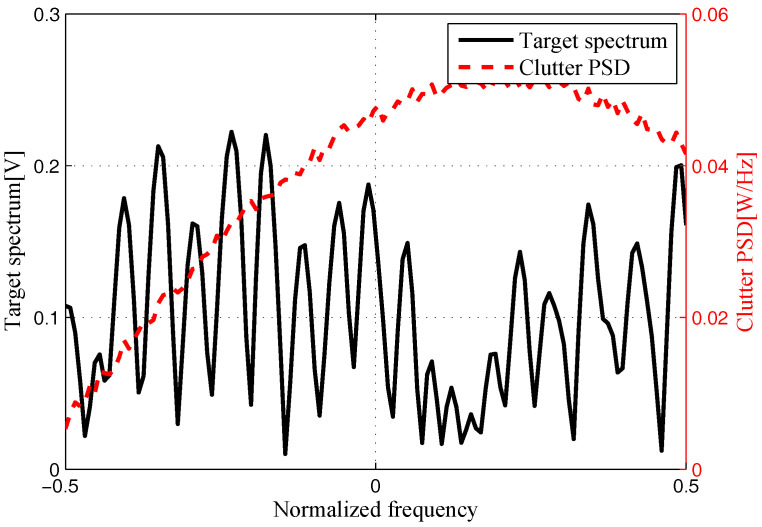
Target and signal-dependent clutter spectra with respect to Radar 1.

**Figure 6:**
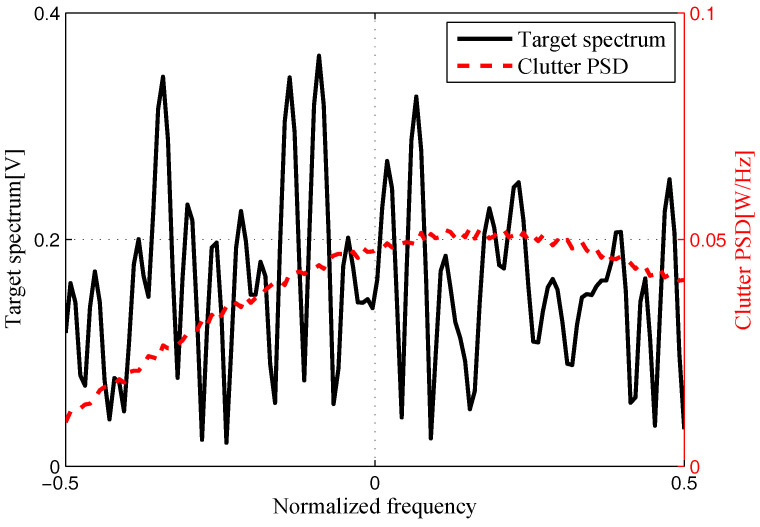
Target and signal-dependent clutter spectra with respect to Radar 2.

**Figure 7:**
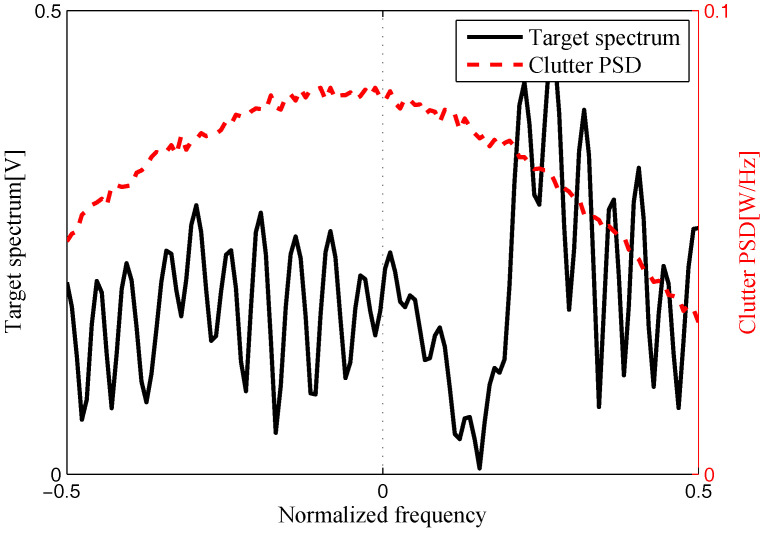
Target and signal-dependent clutter spectra with respect to Radar 3.

**Figure 8:**
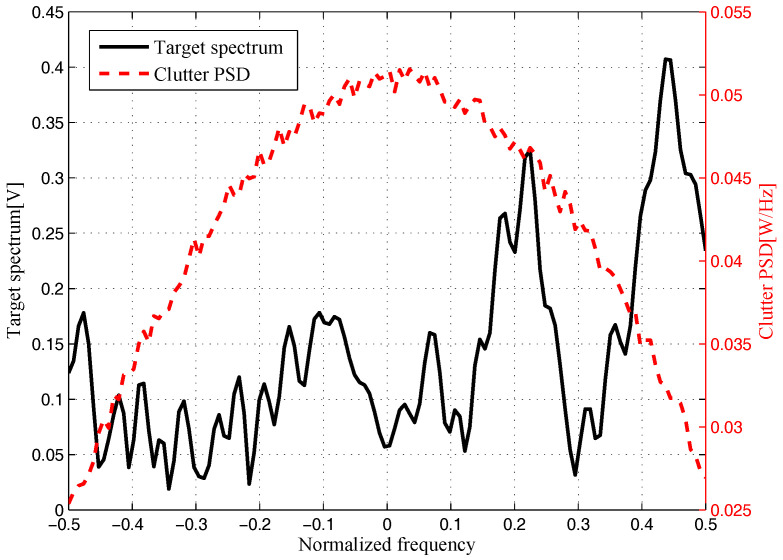
Target and signal-dependent clutter spectra with respect to Radar 4.

**Figure 9:**
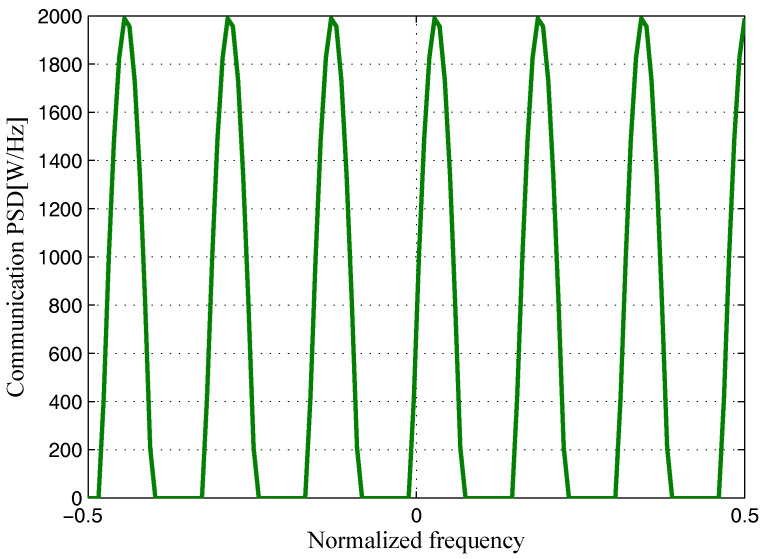
Power spectral density (PSD) of a communication system.

**Figure 10:**
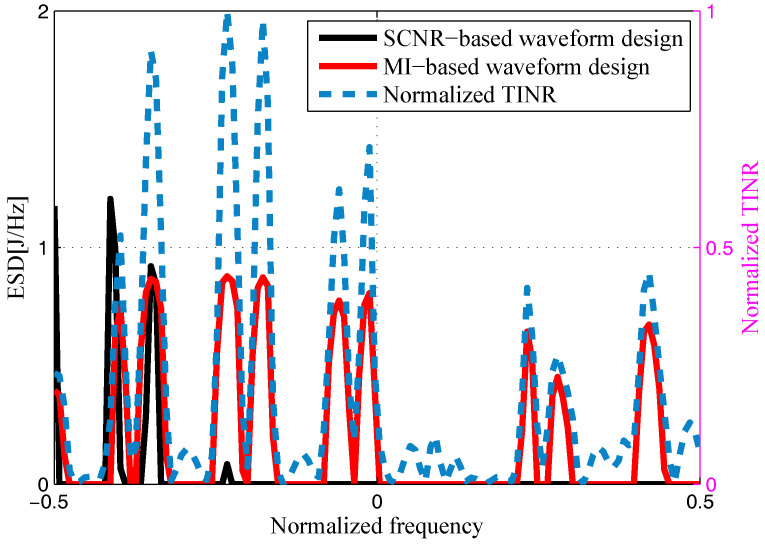
Energy spectral density (ESD) of the resulting Radar 1’s transmit waveform.

**Figure 11:**
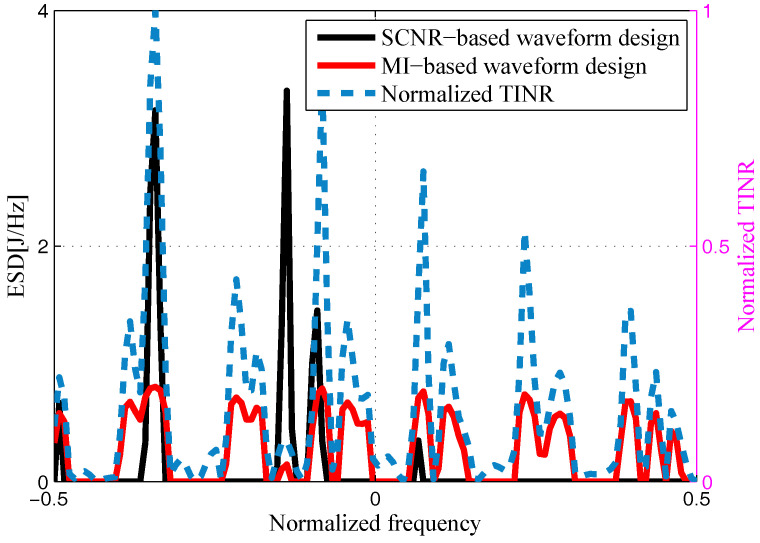
Energy spectral density (ESD) of the resulting Radar 2’s transmit waveform.

**Figure 12:**
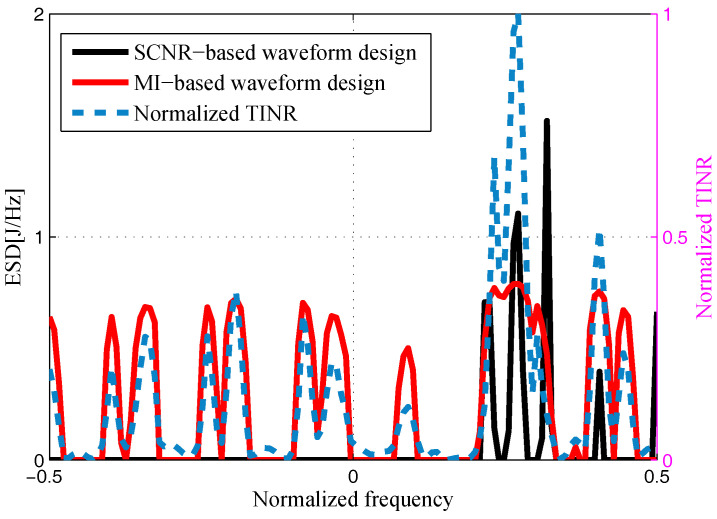
Energy spectral density (ESD) of the resulting Radar 3’s transmit waveform.

**Figure 13:**
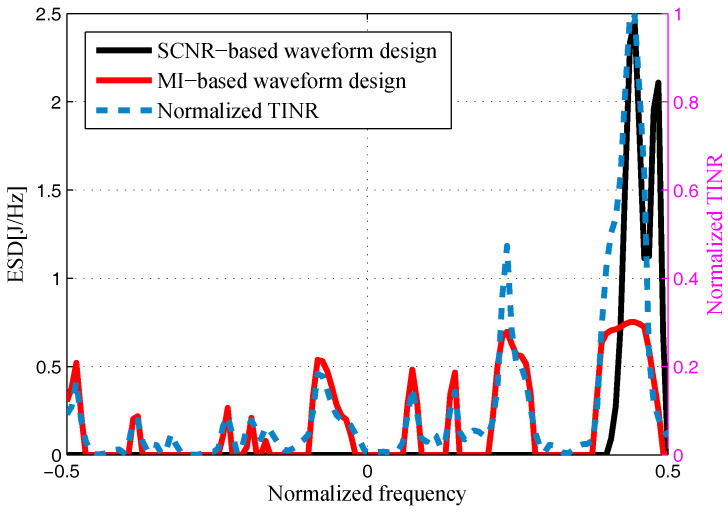
Energy spectral density (ESD) of the resulting Radar 4’s transmit waveform.

**Figure 14:**
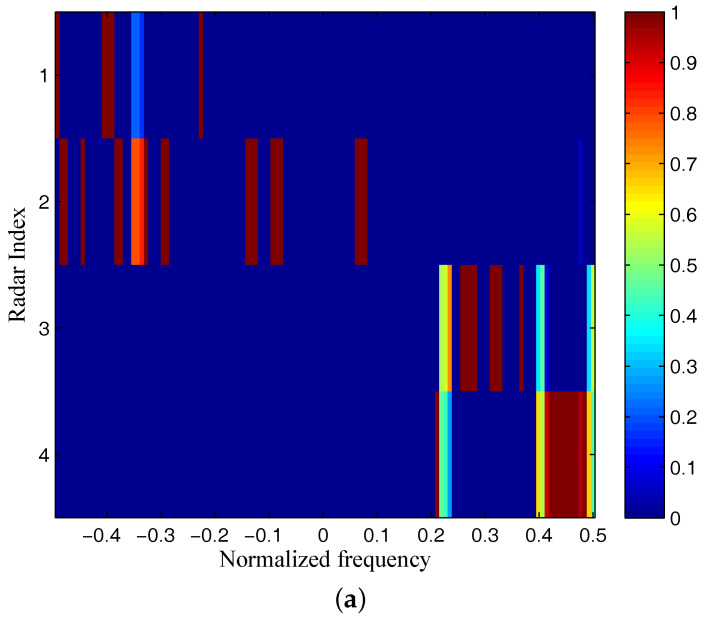

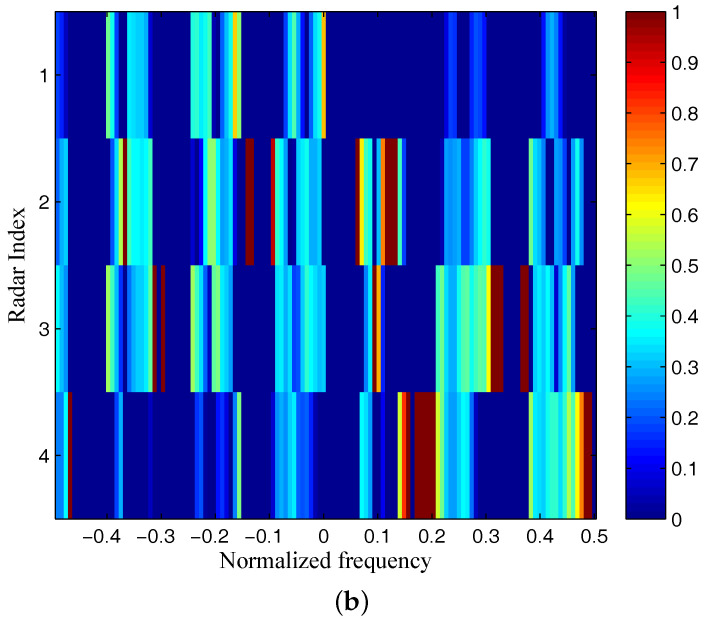
The transmit energy ratio results of DMRS: (**a**) SCNR-based radar waveform design; (**b**) MI-based radar waveform design.

The target and signal-dependent clutter spectra with respect to different radars are shown in Figure 5, Figure 6, Figure 7 and Figure 8, where the clutters characteristics can be estimated by each radar receiver through previous received signals. The PSD of communication signal is illustrated in Figure 9. Figure 10, Figure 11, Figure 12 and Figure 13 depict the optimal radar waveform design results. The transmit energy ratio results employing SCNR- and MI-based optimal radar waveform design strategies are highlighted in Figure 14, which gives insight about the transmit energy allocation for the LPI performance of DMRS, with different colors denoting the ratio of the transmit energy of each radar. Herein, the energy ratio is defined as $\delta_{i}=\frac{|X_{i}{\left(f\right)|}^{2}}{\sum_{i=1}^{N_{t}}{\left|X_{i}\left(f\right)\right|}^{2}}$. For both SCNR- and MI-based optimal radar waveform design strategies, one can notice that the transmit energy allocation is determined by the target spectra and the PSD of communication waveform. Specifically, from Figure 14, we should concentrate more transmit energy for the radar that has a large $|H_{r,i}\left(f\right)|\;\left(\forall i\right)$ and suffers less communication interference power, namely large ${\mathrm{TCNR}}_{i}\left(f\right)\;\left(\forall i\right)$, as shown in Equation . On the other hand, for the radar whose $|H_{r,i}\left(f\right)|$ is weak while the interference power provided by the communication system is strong, we should distribute less energy for the corresponding radar [22,26]. Therefore, one needs to only allocate all the waveform energy in the frequency component achieves the maximum value of ${\mathrm{TCNR}}_{i}\left(f\right)$.

In order to minimize the total transmitted energy for a predetermined performance requirement, the SCNR-based optimal radar waveform design scheme is formed by a water-filling policy, which only places the minimum energy over the dominant frequency components with the largest ${\mathrm{TCNR}}_{i}\left(f\right)$. However, the MI-based optimal radar waveform design scheme allocates the transmit energy over multiple frequency bands. As aforementioned, this is due to the fact that the calculation of MI involves the log computations, which lowers those terms that are related to the transmission energy over frequency components that have large coefficients and small communication interference power. In addition, it is worth mentioning that the proposed optimal radar waveform design approaches place most of the energy at the edges of the communication signal mainlobe by taking advantage of the nulls of the communication bandwidth while minimizing the effects to the communication system.

### 4.3. Comparison of LPI Performance

**Figure 15:**
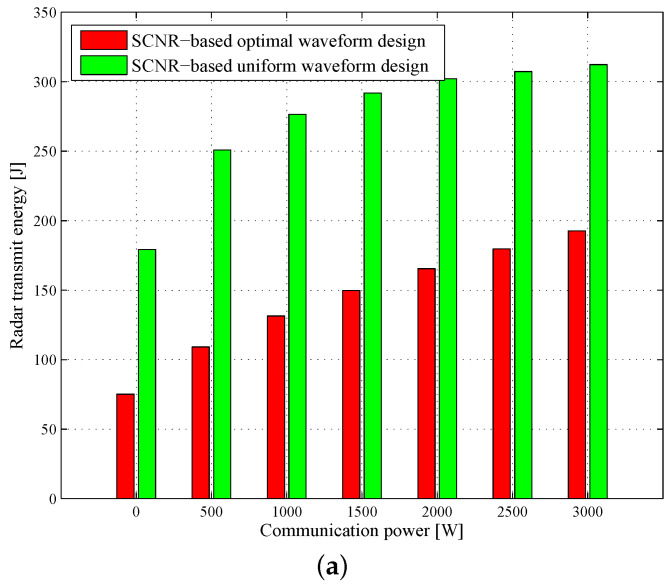

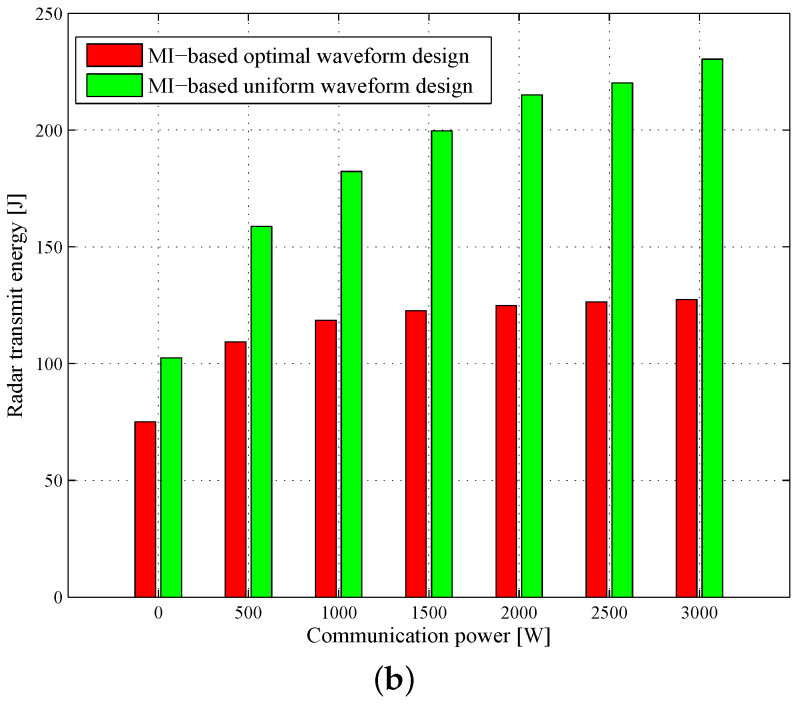
Comparisons of radar transmit energy employing different methods: (**a**) SCNR-based radar waveform design; (**b**) MI-based radar waveform design.

Furthermore, to examine the LPI performance of the SCNR- and MI-based radar waveforms under different communication transmit power, we use the same values for all parameters as in the previous simulation except that the communication transmit power budget changes from 0W to 3000W. Figure 15 shows and compares the radar transmit energy by employing different waveform design methods for different communication power, where Monte Carlo simulations with $10^{4}$ independent trials are conducted to get an average performance. As expected, the radar transmitted energy is increased as the communication power goes up. In addition, it is observed that the proposed SCNR- and MI-based optimal radar waveform design strategies enable us to reduce the radar transmitted energy to 41.8–73.2% of those obtained by uniform waveforms, where the uniform waveforms spread the transmit energy uniformly in the whole frequency band. Overall, the results highlight that the LPI performance of DMRS coexisting with a wireless communication system can be significantly improved by exploiting the proposed radar waveform design schemes.

Based on these results, it can be concluded that DMRS can coexist with communication systems and achieve better LPI performance than traditional pulsed radars, while guaranteeing a specified target detection/estimation performance. Such significant advantage is introduced by the optimal radar waveform design strategies as discussed in Section 2. Moreover, the communication system works as if no radar is present during the radar transmit time by utilizing the proposed optimal radar waveform design strategies, which has been shown in [23] and is omitted here for brevity. The resulting symbol error rate (SER) of communication system is very close to theoretical noise-only SER.

## 5. Conclusions

In this paper, we have investigated the coexistence of a distributed multiple-radar and a wireless communication system by sharing a common carrier frequency. The idea was to design the transmission waveform of each radar to minimize the total transmitted energy subject to a desired target detection/characterization performance requirement, while trying not to interfere with friendly communications. The SCNR- and MI-based optimal radar waveform design strategies were formulated and solved analytically. The proposed radar waveform design schemes have been evaluated via extensive simulations. To be specific, we have seen that DMRS achieves better LPI performance than traditional pulsed radars. Our simulation results suggest that DMRS can coexist with communication systems and achieve better LPI performance than traditional pulsed radars, while saving up to 58.2% in transmit energy. We should notice that the proposed LPI-based radar waveform design methods can also be applied to traditional monostatic radar, which is a special case of DMRS for $N_{t}=1$. In future work, we will address the noise radar waveform design to further enhance the LPI performance of DMRS. 

## Figures and Tables

**Table 1:** Coexisting distributed multiple-radar and communication systems parameters.

Parameter	Value	Parameter	Value
$G_{t,i}\left(\forall i\right)$	40dB	$G_{r,i}\left(\forall i\right)$	50dB
$G_{r,i}^{\prime }\left(\forall i\right)$	−40dB	$G_{s}$	0dB
W	512MHz	$S_{\mathrm{nn},i}\left(f\right)$	$1.66\times 10^{-14}\;W/\mathrm{Hz}$

## References

[1] Fisher E., Haimovich A., Blum R.S., Cimini L.J., Chizhik D., Valenzuela R. (2006). Spatial diversity in radars—Models and detection performance. IEEE Trans. Signal Process..

[2] Haimovich A.M., Blum R.S., Cimini L.J. (2008). MIMO radar with widely separated antennas. IEEE Signal Process. Mag..

[3] Yan J.K., Liu H.W., Pu W.Q., Zhou S.H., Liu Z., Bao Z. (2016). Joint beam selection and power allocation for multiple target tracking in netted colocated MIMO radar system. IEEE Trans. Signal Process..

[4] Aubry A., Maio A.D., Huang Y., Piezzo M., Farina A. (2015). A new radar waveform design algorithm with improved feasibility for spectral coexistence. IEEE Trans. Aerosp. Electron. Syst..

[5] Wang H.Y., Johnson J.T., Baker C.J. (2017). Spectrum sharing between communications and ATC radar systems. IET Radar Sonar Navig..

[6] Li B., Petropulu A. (2017). Joint transmit designs for co-existence of MIMO wireless communications and sparse sensing radars in clutter. IEEE Trans. Aerosp. Electron. Syst..

[7] Labib M., Reed J.H., Martone A.F., Zaghloul A.I. A game-theoretic approach for radar and LTE systems coexistence in the unlicensed band. Proceedings of the 2016 USNC-URSI Radio Science Meeting.

[8] Giorgetti A., Chiani M., Win M.Z. (2005). The effect of narrowband interference on wideband wireless communication systems. IEEE Trans. Commun..

[9] Aubry A., Carotenuto V., De Maio A. (2016). Forcing multiple spectral compatibility constraints in radar waveforms. IEEE Signal Process. Lett..

[10] Aubry A., Carotenuto V., De Maio A., Farina A., Pallotta L. (2016). Optimization theory-based radar waveform design for spectrally dense environments. IEEE Aerosp. Electron. Syst. Mag..

[11] Pinto P.C., Giorgetti A., Win M.Z., Chiani M. (2009). A stochastic geometry approach to coexistence in heterogeneous wireless networks. IEEE J. Sel. Areas Commun..

[12] ElSawy H., Sultan-Salem A., Alouini M.S., Win M.Z. (2017). Modeling and analysis of cellular networks using stochastic geometry: A tutorial. IEEE Commun. Surv. Tutor..

[13] Gogineni S., Rangaswamy M., Nehorai A. Multi-modal OFDM waveform design. Proceedings of the IEEE Radar Conference (RadarConf).

[14] Turlapaty A., Jin Y.W. A joint design of transmit waveforms for radar and communication systems in coexistence. Proceedings of the IEEE Radar Conference (RadarConf).

[15] Bica M., Koivunen V. Delay estimation method for coexisting radar and wireless communication systems. Proceedings of the IEEE Radar Conference (RadarConf).

[16] Chiriyath A.R., Paul B., Jacyna G.M., Bliss D.W. (2016). Inner bounds on performance of radar and communications co-existence. IEEE Trans. Signal Process..

[17] Zheng L., Lpos M., Wang X.D., Grossi E. (2017). Joint design of overlaid communication systems and pulsed radars. IEEE Trans. Signal Process..

[18] Shi C.G., Wang F., Zhou J.J., Zhang H. Security information factor based low probability of identification in distributed multiple-radar system. Proceedings of the IEEE International Conference on Acoustics, Speech and Signal Processing (ICASSP).

[19] Zhang Z.K., Tian Y.B. (2016). A novel resource scheduling method of netted radars based on Markov decision process during target tracking in clutter. EURASIP J. Adv. Signal Process..

[20] Shi C.G., Zhou J.J., Wang F. LPI based resource management for target tracking in distributed radar network. Proceedings of the IEEE Radar Conference (RadarConf).

[21] Shi C.G., Wang F., Sellathurai M., Zhou J.J. (2017). Low probability of intercept based multicarrier radar jamming power allocation for joint radar and wireless communications systems. IET Radar Sonar Navig..

[22] Shi C.G., Wang F., Sellathurai M., Zhou J.J., Zhang H. (2016). Robust transmission waveform design for distributed multiple-radar systems based on low probability of intercept. ETRI J..

[23] Romero R.A., Shepherd K.D. (2015). Friendly spectrally shaped radar waveform with legacy communication systems for shared access and spectrum management. IEEE Access.

[24] Huang K.W., Bica M., Mitra U., Koivunen V. Radar waveform design in spectrum sharing environment: Coexistence and cognition. Proceedings of the IEEE Radar Conference (RadarConf).

[25] Bica M., Huang K.W., Mitra U., Koivunen V. Opportunistic radar waveform design in joint radar and cellular communication systems. Proceedings of the IEEE Global Communications Conference (GLOBECOM).

[26] Bica M., Huang K.W., Koivunen V., Mitra U. Mutual information based radar waveform design for joint radar and cellular communication systems. Proceedings of the IEEE International Conference on Acoustics, Speech and Signal Processing (ICASSP).

[27] Shi C.G., Salous S., Wang F., Zhou J.J. (2016). Low probability of intercept based adaptive radar waveform optimization in signal dependent clutter for joint radar and cellular communication systems. EURASIP J. Adv. Signal Process..

[28] Shi C.G., Wang F., Sellathurai M., Zhou J.J., Salous S. (2018). Power minimization based robust OFDM radar waveform design for radar and communication systems in coexistence. IEEE Trans. Signal Process..

[29] Romero R.A., Bae J., Goodman N.A. (2011). Theory and application of SNR and mutual information matched illumination waveforms. IEEE Trans. Aerosp. Electron. Syst..

[30] Bell M.R. (1993). Information theory and radar waveform design. IEEE Trans. Inf. Theory.

[31] Chen Y., Nijsure Y., Chew Y.H., Ding Z., Boussakta S. (2013). Adaptive distributed MIMO radar waveform optimization based on mutual information. IEEE Trans. Aerosp. Electron. Syst..

[32] Shao H., Beaulieu N.C. (2011). Direct sequence and time-hopping sequence designs for narrowband interference mitigation in impulse radio UWB systems. IEEE Trans. Commun..

[33] Giorgetti A. Interference mitigation technique by sequence design in UWB cognitive radio. Proceedings of the 3rd International Symposium on Applied Sciences in Biomedical and Communication Technologies (ISABEL 2010).

[34] Lin F.Y., Liu J.M. (2004). Ambiguity functions of laser-based chaotic radar. IEEE J. Quantum Electron..

[35] Kay S. (2007). Optimal radar signal for detection in clutter. IEEE Trans. Aerosp. Electron. Syst..

[36] Wang L.L., Wang H.Q., Wong K.K., Brennan P.V. (2014). Minimax robust jamming techniques based on signal-to-interference-plus-noise ratio and mutual information. IET Commun..

